# 

**Published:** 1886-09

**Authors:** 


### CONTENTS:

ARTICLE I—Southern Dental Association.... 193
" II—Pyorrhœa Alveolaris. By Alfred R. Starr, M. D., D. D. S.... 221
" III—Dental Jurisprudence. By Richard Grady, D. D. S .... 228

### EDITORIAL, ETC.

The University of Maryland, Dental Department.... 232
Dr. William Herbst.... 233

### BIBLIOGRAPHICAL.

The American System of Dentistry.... 236

### MONTHLY SUMMARY.

Aluminium Bronze in Medicine and Dentistry.... 237
Boric Acid and Affections of the Mouth.... 238
Convulsions, Etc.... 240

